# Genome wide association studies in presence of misclassified binary responses

**DOI:** 10.1186/1471-2156-14-124

**Published:** 2013-12-26

**Authors:** Shannon Smith, El Hamidi Hay, Nourhene Farhat, Romdhane Rekaya

**Affiliations:** 1 Department of Animal and Dairy Science, The University of Georgia, Athens, GA, USA; 2 Department of Statistics, The University of Georgia, Athens, GA, USA; 3 Institute of Bioinformatics, The University of Georgia, Athens, GA, USA; 4 PCOM, Suwanee, Athens, GA, USA

**Keywords:** Misclassification, Genome wide association, Discrete responses

## Abstract

**Background:**

Misclassification has been shown to have a high prevalence in binary responses in both livestock and human populations. Leaving these errors uncorrected before analyses will have a negative impact on the overall goal of genome-wide association studies (GWAS) including reducing predictive power. A liability threshold model that contemplates misclassification was developed to assess the effects of mis-diagnostic errors on GWAS. Four simulated scenarios of case–control datasets were generated. Each dataset consisted of 2000 individuals and was analyzed with varying odds ratios of the influential SNPs and misclassification rates of 5% and 10%.

**Results:**

Analyses of binary responses subject to misclassification resulted in underestimation of influential SNPs and failed to estimate the true magnitude and direction of the effects. Once the misclassification algorithm was applied there was a 12% to 29% increase in accuracy, and a substantial reduction in bias. The proposed method was able to capture the majority of the most significant SNPs that were not identified in the analysis of the misclassified data. In fact, in one of the simulation scenarios, 33% of the influential SNPs were not identified using the misclassified data, compared with the analysis using the data without misclassification. However, using the proposed method, only 13% were not identified. Furthermore, the proposed method was able to identify with high probability a large portion of the truly misclassified observations.

**Conclusions:**

The proposed model provides a statistical tool to correct or at least attenuate the negative effects of misclassified binary responses in GWAS. Across different levels of misclassification probability as well as odds ratios of significant SNPs, the model proved to be robust. In fact, SNP effects, and misclassification probability were accurately estimated and the truly misclassified observations were identified with high probabilities compared to non-misclassified responses. This study was limited to situations where the misclassification probability was assumed to be the same in cases and controls which is not always the case based on real human disease data. Thus, it is of interest to evaluate the performance of the proposed model in that situation which is the current focus of our research.

## Background

Misclassification of dependent variables is a major issue in many areas of science that can arise when indirect markers are used to classify subjects or continuous traits are treated as categorical [1]. Binary responses are typically subjective measurements which can lead to error in assigning individuals to relevant groups in case–control studies. Many quantitative traits have precise guidelines for measurements but in qualitative diagnosis different individuals will understand conditions in their own way [2]. Some disorders require structured evaluations but these can be time consuming and very costly and not readily available for all patients [3]. This sometimes requires clinicians to use heuristics rather than following strict diagnostic criteria [4], leading to diagnoses based on personal opinions and experience. It was found that physicians will disagree with one another one third of the time as well as with themselves (on later review) one fifth of the time. This lack of consistency leads to large variation and error [5,6].

Researchers indicated that there is a common assumption under most approaches that disorders can be distinguished without error which is seldom the case [7]. For instance, a longitudinal study was carried out over 10 years where 15% of subjects initially diagnosed with bipolar disorder were re-diagnosed with schizophrenia, whereas 4% were reclassified in the opposite direction [8]. Reports have shown an error rate of more than 5-10% for some discrete responses [9,10]. In some instances, these rates have proven to be significantly higher. The frequency of medical misdiagnosis and clinical errors has reached error rates as high as 47% as documented in several autopsy studies [11]. Error rates in clinical practices have shown to be higher than perceptual specialties [12], but still these areas have demonstrated high rates as well. In radiology areas, failure to detect abnormalities when they were present (false negative) ranged between 25-30%, and when the cases were normal but incorrectly diagnosed as diseased (false positive) ranged between 1.5-2% [13]. Some stated that these errors are not due to failure of not showing on film but due to perceptual errors [14]. These findings are similar to recent published studies [3,6,15,16].

Unfortunately, finding these errors in clinical data is not trivial. Even in the best case scenario when well-founded suspicion exists about a sample, re-testing is often not possible and the best that could be done is to remove the sample leading to power reduction. Recently, several research groups [17-19] have proposed using single nucleotide polymorphisms (SNPs) to evaluate the association between discrete responses and genomic variations. Genome-wide association studies (GWAS) provide researchers with the opportunity of discovering genomic variations affecting important traits such as diseases in humans, and production and fitness responses in livestock and plant species. Several authors have indicated that the precision and validity of GWAS relies heavily on the accuracy of the SNP genotype data as well as the certainty of the response variable [20-25]. Thus, analyzing misclassified discrete data without correcting or accounting for these errors may cause algorithms to select polymorphisms with little or no predictive ability. This could lead to varying and even contradictory conclusions. In fact, it was reported that only 6 out of 600 gene-disease associations reported in the literature were significant in more than 75% of the studies published [26]. In majority of cases, heterogeneity, population stratification, and potential misclassification in the discrete dependent variables were at the top of the list of potential reasons for these inconsistent results [22,27-30].

In supervised learning, if individuals are wrongly assigned to subclasses, false positive and erroneous effects will result if these phenotypes are used when trying to identify which markers or genes can distinguish between disease subclasses. Researchers carried out a study of misclassification using gene expression data with application to human breast cancer [31]. They looked at the influence of misclassification on gene selection. It was found that even when only one sample is misclassified, 20% of the most significant genes were not identified. Further results showed that with misclassification rates between 3-13%, there could be unfavorable effect on detecting the most significant genes for disease classification. Furthermore, if some genes are identified as significant while misclassification is present, this will lead to the inability to replicate the results due to the fact it is only relevant to the specific data.

To overcome these issues it would be advantageous to develop a statistical model that is able to account for misclassification in discrete responses. There have been several approaches proposed on how to handle misclassification. Researchers have suggested Bayesian methods [32-34], some described a latent Markov model for longitudinal binary data [35], others proposed marginal analysis methods [36], and some considered two-state Markov models with misclassified responses [37,38].

In 2001, a Bayesian approach was proposed for dealing with misclassified binary data [34]. This procedure, with the use of Gibbs sampling, “made the analysis of binary data subject to misclassification tractable”. It was concluded that failure to account for errors in responses results in adverse effects related to the parameters of interest including genetic variance. The analysis was applied to simulated cow fertility data and was later implemented with the use of real data which resulted in similar findings [10,31]. One study found considering a potential for misdiagnosis in the data could increase prediction power by 25% [10]. To extend their ideas we simulated a typical case–control study to measure and understand the effects of misclassification on GWAS using a threshold model and misclassification algorithm. Three analyses were conducted: (M1) the true data was analyzed with a standard threshold model; (M2) the noisy (5% and 10% miscoding) data analyzed with standard threshold model ignoring miscoding; (M3) the noisy data analyzed with threshold model with probability of being miscoded (π) included.

## Methods

### Detecting discrete phenotype errors

Let **y** = (*y*_1_, *y*_2_, …, *y*_*n*_) ', be a vector of binary responses observed for *n* individuals and genotypes for a set of SNPs are available for each. The problem is being able to link these responses to the measured genotypes when miscoding or misclassification of the binary status is present in the samples. Specifically, the observed binary data is a “contaminated” sample of a real unobserved data **r** = (*r*_1_, *r*_2_, …, *r*_*n*_) ', where each r_i_ is the outcome of an independent Bernoulli trial with a success probability, p_i_ specific to each response. Misclassification then occurs when some of the r_i_ become switched. Assuming this error happens with probability *π*, the joint probability of observing the actual data given the unknown parameters is:

$$\begin{array}{l} p\left(y|p,\pi \right)=\prod_{i=1}^{n}[p_{i}(1-\pi )+(1-p_{i})\pi ]{}^{y_{i}}{\left[p_{i}\pi +\left(1-p_{i}\right)\left(1-\pi \right)\right]}^{\left(1-y_{i}\right)}\\ \;=\prod_{i=1}^{n}{\left(q_{i}\right)}^{y_{i}}(1-q_{i}){}^{\left(1-y_{i}\right)} \end{array}$$

With *q*_*i*_ = *p*_*i*_(1 - *π*) + (1 - *p*_*i*_)*π*

The success probability for each observation (*p*_*i*_) is then modeled as a function of the unknown vector of parameters *β*, which in this case is the vector of SNP effects. Assuming conditional independence, the conditional distribution of the true data, **r**, given *β* becomes:

$$p\left(r|\beta \right)=\prod_{i=1}^{n}{\left[p_{i}\left(\beta \right)\right]}^{r_{i}}{\left[1-p_{i}\left(\beta \right)\right]}^{\left(1-r_{i}\right)}$$

where *p*_*i*_(*β*) indicates that *p*_*i*_ is a function of the vector of parameters *β*.

Let **α** = [*α*_1_, *α*_2_, …, *α*_*n*_] ', where*α*_*i*_ is an indicator variable for observation *i* that takes the value of one (α_i_ = 1) if r_i_ is switched and 0 otherwise. Supposing each α_i_ is a Bernoulli trial with success probability π, then $p\left(\alpha_{i}|\pi \right)=\pi^{\alpha_{i}}{\left(1-\pi \right)}^{\left(1-\alpha_{i}\right)}$, the joint distribution of **α** and **r** given *β* and π can be written as:

(1) $$p\left(\alpha ,r|\pi ,\beta \right)=\prod_{i=1}^{n}\pi^{\alpha_{i}}{\left(1-\pi \right)}^{1-\alpha_{i}}{\left[p_{i}\left(\beta \right)\right]}^{r_{i}}{\left[1-p_{i}\left(\beta \right)\right]}^{\left(1-r_{i}\right)}$$

Furthermore, the true unobserved binary data could be written as a function of the observed contaminated binary responses and the vector **α** as:

(2) $$r_{i}=\left(1-\alpha_{i}\right)y_{i}+\alpha_{i}\left(1-y_{i}\right)$$

Notice that when *α*_*i*_ = 0(no switching), the formula in (2) reduces to *r*_*i*_ = *y*_*i*_

Using the relationship in (2), the joint probability distribution of **α** and **y** given **β** and **π** becomes:

$$\begin{array}{l} p\left(\alpha ,y|\pi ,\beta \right)=\prod_{i=1}^{n}\pi^{\alpha_{i}}{\left(1-\pi \right)}^{1-\alpha_{i}}{\left[p_{i}\left(\beta \right)\right]}^{\left(1-\alpha_{i}\right)y_{i}+\alpha_{i}\left(1-y_{i}\right)}\\ \;\times {\left[1-p_{i}\left(\beta \right)\right]}^{1-\left(1-\alpha_{i}\right)y_{i}-\alpha_{i}\left(1-y_{i}\right)} \end{array}$$

To finalize the Bayesian formulation, the following priors were assumed to the unknown parameters in the model

(3) $$\beta \sim U\;\left[\beta_{\min},\beta_{\max}\right]\;\mathrm{and}\;\pi |a,b\sim \mathrm{Beta}\left(a,b\right)$$

where **β**_min_, **β**_max_, *a* and *b* are known hyper-parameters. In our case *a* and *b* were set heuristically to 1 and 4, respectively, in order to convey limited prior information. From our previous experience, these values for the hyper-parameters have little effects on the posterior inferences and the results were similar to those obtained using a flat prior for π. Obviously, the effect of these hyper-parameters depends on the magnitude of n (number of observations). Thus, a special attention has to be placed on specifying these parameters when using small samples and a sensitivity analysis is recommended. For the SNP effects, **β**_min_ and **β**_max_ were set to -100 and 100 respectively conveying, thus, a very vague bounded prior. With real data, it is often the case that the number of SNPs is much larger than the number of observations. In such scenario, an informative prior is needed to make the model identifiable and often a normal prior is assumed.

The resulting joint posterior density of π, **β**, **α** is:

(4) $$\begin{array}{l} p\left(\beta ,\alpha ,\pi |y\right)\propto \prod_{i=1}^{n}{\left[p_{i}\left(\beta \right)\right]}^{\left(1-\alpha_{i}\right)y_{i}+\alpha_{i}\left(1-y_{i}\right)}{\left[1-p_{i}\left(\beta \right)\right]}^{\left[(1-\left(1-\alpha_{i}\right)y_{i}-\alpha_{i}\left(1-y_{i}\right)\right]}\\ \;\times \prod_{i=1}^{n}\pi^{\alpha_{i}}{\left(1-\pi \right)}^{\left(1-\alpha_{i}\right)}p(\pi |a,b) \end{array}$$

Implementation of the model in (4) could be facilitated greatly by using a data augmentation algorithm as described by fellow researchers [33]. It consists in assuming the existence of an unknown continuous random variable, *l*_*i*_, that relates to the binary responses through the following relationship:

$$y_{i}=\left\{\begin{array}{c} 1\;\mathrm{if}\;l_{i}>T\\ 0\;\mathrm{otherwise} \end{array}\right)$$

where *T* is an arbitrary threshold value.

The model at the liability scale could be written as:

(5) $$l_{i}=\mu +\sum_{j=1}^{p}x_{\mathrm{ij}}\beta_{j}+e_{i}$$

where *μ *is the overall mean, *x*_*ij*_ is the genotype for SNP *j* for individual *i, β*_*j*_is the effect of SNP *j (j = 1,1000)* and *e*_*i*_is the residual term. To make the model in (4) identifiable, two restrictions are needed. It was assumed that the T = 0 and *var*(*e*_*i*_) = 1.

At the liability scale and using the prior distributions specified in (3), the full conditional distributions needed for a Bayesian implementation of the model via Gibbs sampler are in closed form being normal for the position parameters [34,39,40] and a binomial distribution for *α*_*i*_

$$\begin{array}{l} p\left(\alpha_{i}|\beta ,\pi ,\alpha_{-i},y\right)\propto {\left[p_{i}\left(\beta \right)\right]}^{\left(1-\alpha_{i}\right)y_{i}+\alpha_{i}\left(1-y_{i}\right)}{\left[1-p_{i}\left(\beta \right)\right]}^{\left(1-\left(1-\alpha_{i}\right)y_{i}-\alpha_{i}\left(1-y_{i}\right)\right)}]\\ \;\times \pi^{\alpha_{i}}{\left(1-\pi \right)}^{\left(1-\alpha_{i}\right)} \end{array}$$

where α_-i_ is vector **α** without α_i_.

For the misclassification probability, its conditional distribution is proportional to

$$p\left(\pi |\beta ,\alpha ,y\right)\propto \prod_{i=1}^{n}\pi^{\alpha_{i}}{\left(1-\pi \right)}^{\left(1-\alpha_{i}\right)}p\left(\pi |a,b\right)$$

Hence, π is distributed as *Beta*(*a* + ∑ *α*_*i*_, *b* + *n* - ∑ *α*_*i*_) with ∑ *α*_*i*_ is the total number of misclassified (switched) observations.

Given *α* and *π*, the conditional distributions of *μ*, *β* and the vector of liabilities, *l*, are easily derived:

$$p\left(\mu |\beta ,\pi ,\alpha ,l,y\right)\sim N\;\left(\frac{\sum_{i=1}^{n}\left(y_{i}-\sum_{j=1}^{p}x_{\mathrm{ij}}\beta_{j}\right)}{n},\frac{1}{n}\right)$$

where n = 2000 is the number of data points.

For each element in the vector *β*

$$p\left(\beta_{j}|\mu ,\beta_{-j},\pi ,\alpha ,l,y\right)\sim N\left({\hat{\beta }}_{j},{\left(x_{j}^{'}x_{j}\right)}^{-1}\right)$$

where ${\hat{\beta }}_{j}={\left(x_{j}^{'}x_{j}\right)}^{-1}x_{j}^{'}\left(y-1_{n}\mu -\mathrm{X\beta}\right)$ with *x*_*j*_ is a column vector of genotypes for SNP *j*, **X** is an *nxp* matrix of SNP genotypes with the *j*^th^ row and column set to zero and *β*_-*j*_is the vector *β* excluding the *j*^th^ position.

For each element in the liability vector,

$$p\left(l_{i}|\mu ,\beta ,\pi ,\alpha ,l_{-i},y\right)\sim \mathrm{TN}\left({\hat{l}}_{i},1\right)$$

This is a truncated normal (TN) distribution to the left if *y*_*i*_ = 1 and to the right if *y*_*i*_ = 0 (Sorensen et al., 1995) where ${\hat{l}}_{i}=\left(\mu +\sum_{j=1}^{p}x_{\mathrm{ij}}\beta_{j}\right)$ and *l*_-*i*_ is the vector *l* excluding the *i*^th^ position.

In all simulation scenarios, the Gibbs sampler was run for a unique chain of 50,000 iterations of which the first 10,000 iterations were discarded as burn-in period. The convergence of the chain was assessed heuristically based on the inspection of the trace plot of the sampling process.

### Simulation

PLINK software [41] was used to simulate a case–control type data sets using the SNP simulation routine. Four simulation scenarios were generated to determine the effects of misclassification of binary status on GWAS. In each scenario, a dataset of 2000 individuals consisting of 1000 cases and 1000 controls was simulated. All individuals were genotyped for 1000 SNPs with minor allele frequencies generated from a uniform distribution between 0.05 and 0.49. SNPs were coded following an additive model (AA = 0, Aa = 1, and aa = 2). Of the 1000 SNPs, 850 SNPs were assumed non-influential and the remaining 150 SNPs were assumed to be associated with the disease status. To mimic realistic scenarios, a series of bins were specified for the 150 influential SNPs to build a spectrum of odds ratios (OR) for disease susceptibility. Two different series of odds ratios were considered. The first group was generated with “moderate” ratios where 25 of the 150 disease associated SNPs were assumed to have an odds ratio of 1:4, 35 with OR of 1:2, and 90 with OR of 1:1.8. The second group was generated using the same distribution except the ratios increased to a more extreme range; 25 with OR 1:10, 35 with OR of 1:4, and 90 with OR of 1:2. Once these parameters were established, PLINK generated a quantitative phenotype based on the disease variants and a random component or error term. Then a median split of that trait was performed thereafter each individual was assigned a binary status. When the “true” binary data were generated as described above, randomly 5 or 10% of the true binary records were miscoded, meaning binary records from cases were switched to controls and vice versa.

Based on the OR distribution (moderate and extreme) and the level of misclassification (5 or 10%), four data sets were generated: 5% misclassification rate and moderate OR (D1); 5% misclassification and extreme OR (D2); 10% misclassification rate and moderate OR (D3); and 10% misclassification rate and extreme OR (D4). For each dataset, 10 replicates were generated.

To further test our proposed method, a more diverse and representative data was simulated using the basic simulation procedure previously indicated. For this second simulation, a dataset consisting of 1800 individuals (1200 controls and 600 cases) was genotyped for 40,000 linked SNPs assuming an additive model. Five hundred SNPs were assumed to be influential with OR set equal to 1:4 (80 SNPs), 1:2 (120 SNPs), and 1:1.8 (300 SNPs). Only the 5% misclassification rate scenario was considered.

## Results and discussion

For all simulation scenarios, the true misclassification probability was slightly underestimated. In fact, the posterior mean (averaged over 10 replicates) was 3 and 6% for D1 and D3, respectively. However for moderate OR, the true misclassification probability values still lie within their respective HPD95% interval indicating the absence of systematic bias (Table 1). As the average odd ratios of influential SNPs increased, the estimated misclassification probability increased to 4 and 7% for D2 and D4, respectively. In both cases the estimated misclassification probability was outside the HPD95% interval however the true value used in the simulation was close to the upper limit. To further test the ability of our procedure to correctly estimate potential misclassification, a null analysis was performed. A true data set (without any misclassification) was analyzed with our proposed model that contemplates misclassification. As expected, the estimated misclassification probability was very close to zero (0.001) indicating, thus, absence of erroneous observations. Across all simulation scenarios, these results indicate the ability of the algorithm to efficiently distinguish between miscoded and correctly coded samples. Similar results were observed when dairy cattle fertility subject to misclassification were analyzed [34] as well as when applied using cancer gene expression data [31].

**Table 1:** **Summary of the posterior distribution of the misclassification probability ( *π *) for the four simulation scenarios (averaged over 10 replicates)**

	**Moderate**^**1**^		**Extreme**	
True *π*	PM^2^	HPD95%	PM	HPD95%
5%	0.03	0.01-0.05	0.04	0.03-0.06
10%	0.06	0.04-0.09	0.07	0.06-0.09

^1^Moderate effects for influential SNPs; ^2^ PM = Posterior mean; ^3^ HPD95% = High probability density interval.

Table 2 presents the correlation between the true and estimated SNP effects, where the true SNP effects were calculated based on the analysis of the true data (M1). As expected, across all simulated scenarios, the use of the proposed methods (M3) to analyze misclassified data has increased the correlation and consequently reduced any potential bias in estimating SNP effects. For instance, when D1 was used, the correlation between true and estimated SNPs effects increased from 0.83 when M2 was used to 0.93 using M3 or an increase of around 12%. As the OR of influential SNPs increased, the difference in predicting the true SNP effects between M2 and M3 increased substantially. In fact, using D2 the accuracy increased by 27% from 0.664 (M2) to 0.843 (M3). The same trend was observed when the probability of misclassification increased from 5 to 10% with an increase in correlation of 0.15 and 0.26 for D3 and D4, respectively. These results indicate not only the superiority of our proposed method compared to a model that ignores potential misclassification (M2) but more importantly is that our methods seems to be robust to the level of misclassification rate or the OR of significant SNPs. Specifically, when the misclassification rate was increased from 5 to 10%, the accuracy of M2 decreased in average by 15% whereas it decreased only by 4% using our method. Furthermore, it is worth highlighting that even on the extreme case scenario (D4), our method still produces consistent results as the correlation between true and estimated SNPs effects was 0.82 (Table 2).

**Table 2:** **Correlation between true**^**1 **^**and estimated SNP effects under four simulation scenarios using noisy data (M2) and the proposed approach (M3)**

	**5%**		**10%**	
	**Moderate**^**2**^	**Extreme**	**Moderate**	**Extreme**
M2	0.828	0.664	0.714	0.558
M3	0.925	0.843	0.864	0.815

^1^True effects were calculated based on analysis of the true data (M1); ^2^Moderate effects for influential SNPs.

Using the data set simulated under a more realistic scenario (imbalance between cases and controls, larger SNP panel) the results were similar in trend and magnitude to those observed using the first four simulations. In fact, the posterior mean of the misclassification probability was 0.04 and the true value (0.05) was well within the HPD95% interval. Furthermore, the correlations between SNP effect estimates using M2 and M3 were 0.54 and 0.70, respectively. This 30% increase in accuracy using M3 indicates a substantial improvement of the model when our proposed method is used. This is of special practical importance as the superiority of the method was maintained with a dataset similar to what is currently used in GWAS.

It is clear that across all simulation scenarios our proposed method (M3) showed superior performance. Accounting for misclassification in the model increases the predictive power by eliminating or at least by attenuating the negative effects caused by these errors, allowing for better estimates of the true SNP effects. This is essential in GWA studies for correctly estimating the proportion of variation in cause of disease associated with SNPs. Complex diseases which are under the control of several genes and genetic mechanisms are moderately to highly heritable [42-44].

**Figure 1:**
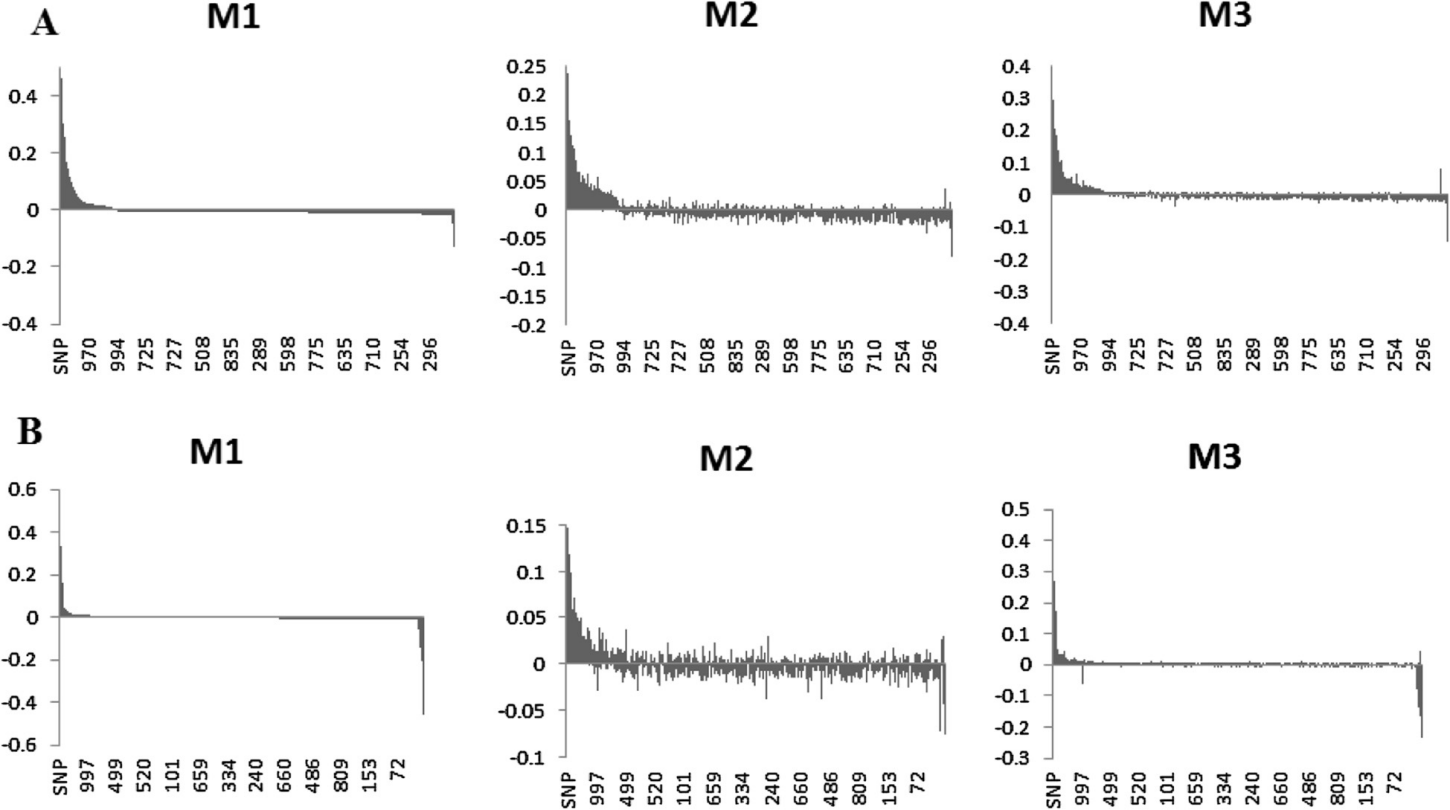
**Distribution of SNP effects for 5% misclassification rate.** The effects are sorted in decreasing order based on estimates using M1 when odds ratios of influential SNPs are moderate **(A)** and extreme **(B)**. M1: misclassification was not present in the data. M2: misclassification was present in the data set but was not addressed. M3: misclassification was addressed using the proposed method.

**Figure 2:**
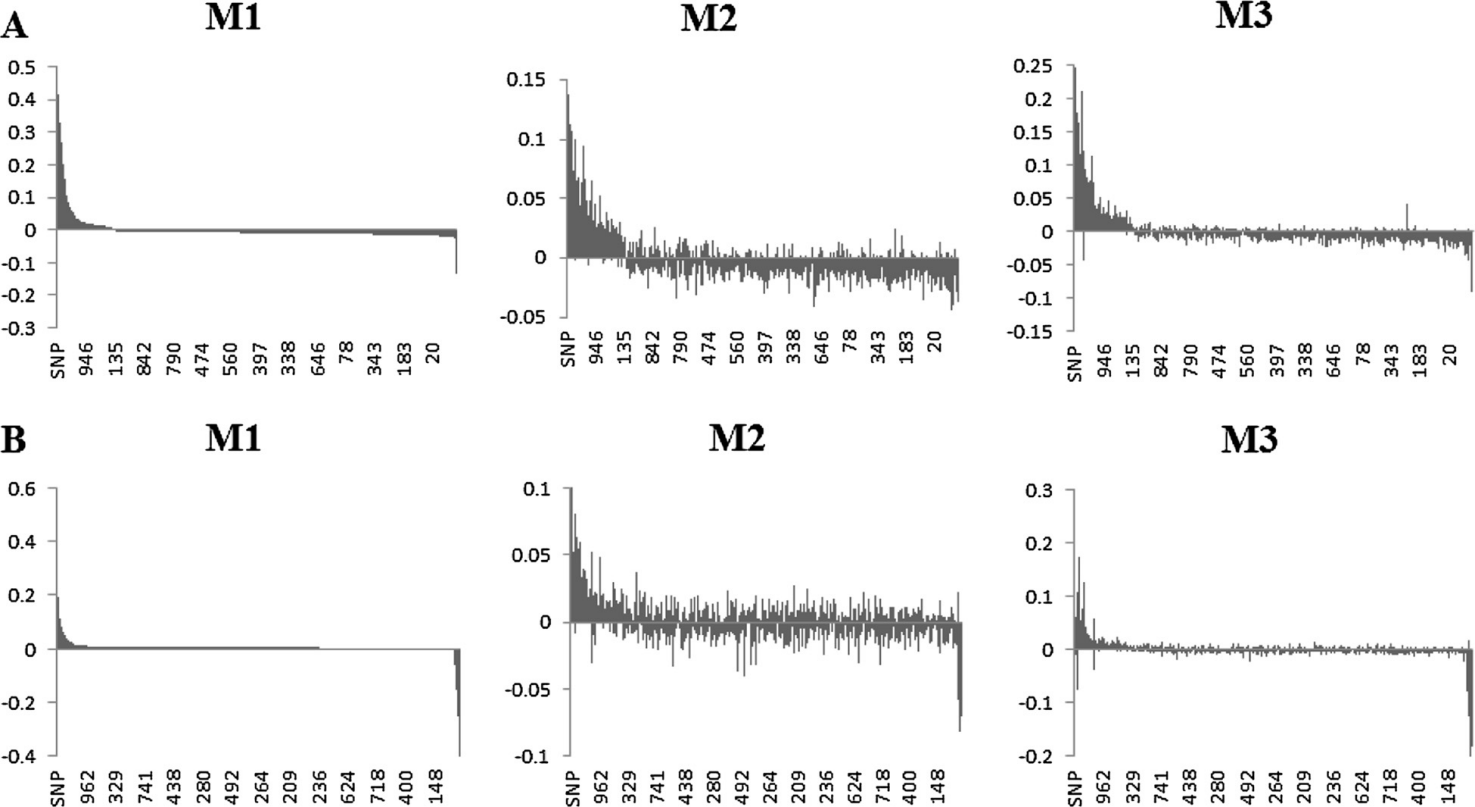
**Distribution of SNP effects for 10% misclassification rate.** The effects are sorted in decreasing order based on estimates using M1 when odds ratios of influential SNPs are moderate **(A)** and extreme **(B)**. M1: misclassification was not present in the data. M2: misclassification was present in the data set but was not addressed. M3: misclassification was addressed using the proposed method.

To further investigate the consequences of misclassification errors on estimating SNP effects we observed the changes in magnitude and the ranking of influential SNPs. As mentioned before the benefits of GWAS lies in its ability to correctly detect polymorphisms associated with a disease. This is driven by how well the model can estimate SNP effects so that the polymorphisms with significant associations will have the largest effects. Figure 1 presents SNP effects ordered in a decreasing order based on their estimates using M1 (no misclassification) for scenarios D1 (Figure 1A) and D2 (Figure 1B). It is clear that in both cases, the M2 method under-performed M3 in estimating the true magnitude and direction of the SNP effects. Even more pronounced results were observed when the misclassification rate was 10% as indicated in Figure 2. In fact, this underestimation effect has been reported as one of the downfalls of GWAS. When approximating SNP effects, there is an estimation error attached to them adding noise and weakening the strength of the effect [45]. In the presence of misclassification this “noise” is inflated which can lead to underestimating the effects of truly significant SNPs. It has been reported this is most severe when the diseases are influenced by numerous risk variants [46].

In addition to an inaccurate estimation of significant SNPs, M2 tends to report non-zero estimates for truly non-influential SNPs, especially under scenario D2, contrary to M1 and M3. For example, under scenario D1, 3 out of the 15 most influential SNPs (top 10%) were not identified by M2 (Table 3). However, only one SNP was not identified using M3. This 20% loss of the most significant polymorphisms exhibited by M2 reduces the power of association. Accounting for potential misclassification as observed with our method aids in reducing false discovery rates which is essential in association studies. Similar results were found under D2 as M2 failed to identify 33% of the top 10% SNPs whereas M3 failed to identify only 13%.

**Table 3:** Number of the top 10% (15 SNPs) most influential SNPs that were correctly identified for all simulation scenarios using the noisy data (M2) and the proposed approach (M3)

	**5%**		**10%**	
	**Moderate**^**1**^	**Extreme**	**Moderate**	**Extreme**
M2	12	10	10	9
M3	14	13	13	12

^1^Moderate and extreme OR for influential SNPs.

**Figure 3:**
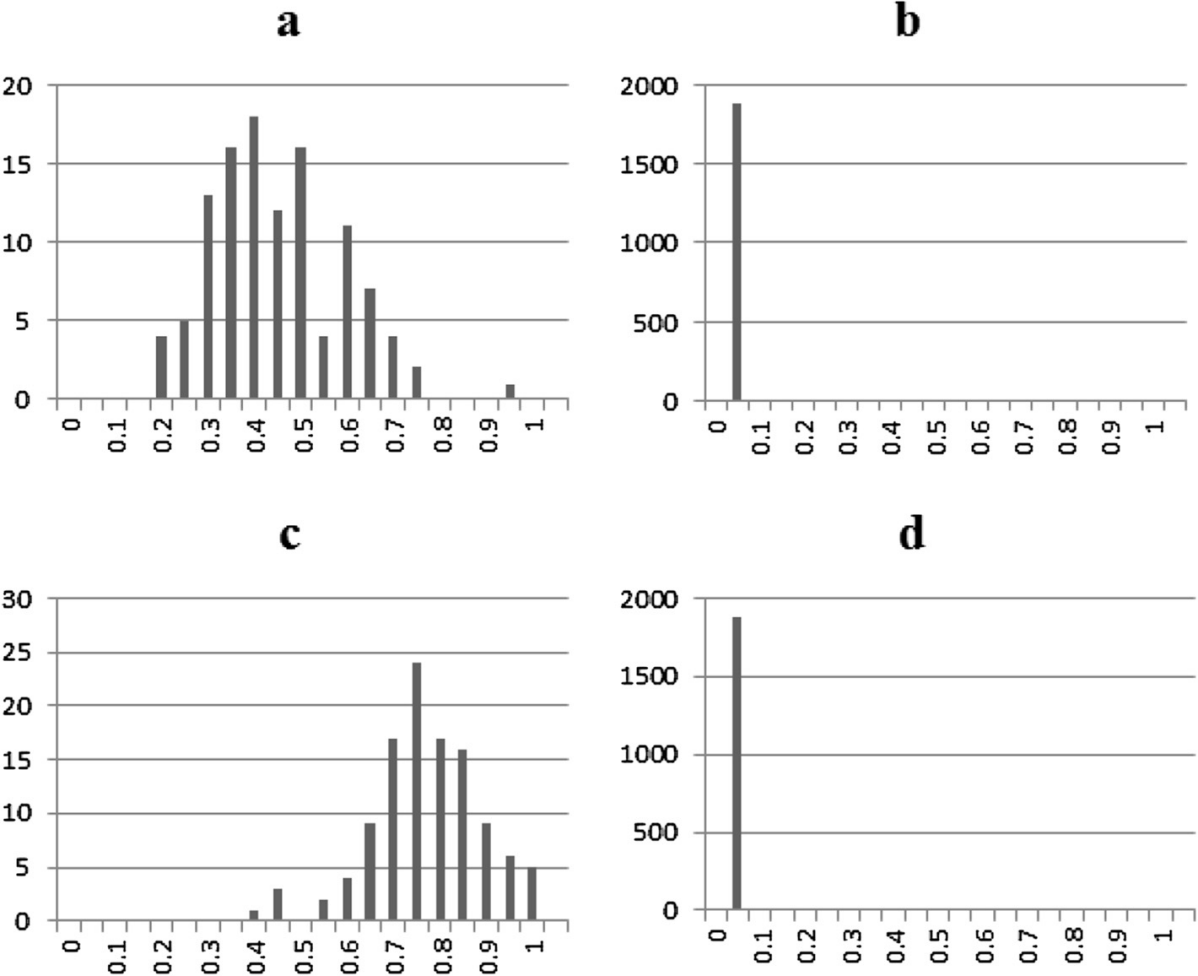
Average posterior misclassification probability for the 113 miscoded observations (a: moderate and c: extreme) and the 1887 correctly coded observations (b: moderate and d: extreme) when the misclassification rate was set to 5%.

**Figure 4:**
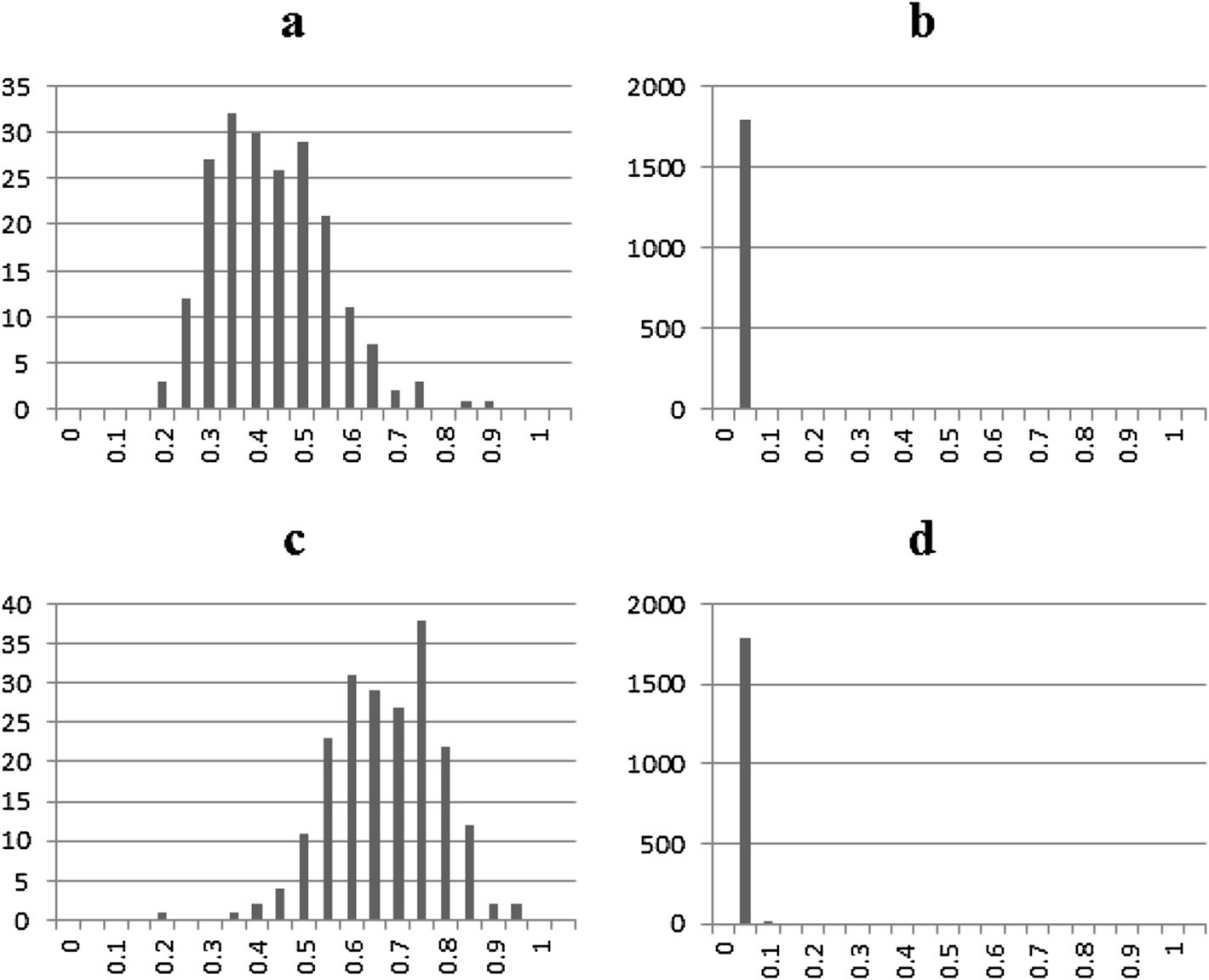
Average posterior misclassification probability for the 205 miscoded observations (a: moderate and c: extreme) and the 1795 correctly coded observations (b: moderate and d: extreme) when the misclassification rate was set to 10%.

To further evaluate the effectiveness of our proposed methods, we looked at its ability of correctly identifying misclassified observations. For that purpose, we calculated the posterior probability of misclassification of each observation in all four scenarios. Figure 3 presents the average posterior misclassification probability for the 113 miscoded observations (Figure 3a and 3c) and the 1887 correctly coded observations (Figure 3b and 3d) when the misclassification rate was set to 5%. For scenario D1, the miscoded group exhibited a higher misclassification probability with a mean of 0.40 compared to a mean of 0.005 for the correctly coded group (Figure 3a and 3b). The lowest misclassification probability observed for the miscoded group was 0.18 far greater than the largest probability calculated for the non-miscoded group which was 0.08 (Figure 3b). This is important as it shows that the algorithm was able to distinguish between the two groups and the miscoded records were detected with a high probability. In fact, when the odd ratios increased (D2) this difference became more sizable, as the averages increased to 0.72 and 0.003 for the miscoded and correctly coded individuals, respectively (Figure 3c and 3d). The same trend held as misclassification increased to 10% as indicated in Figure 4. When D3 (D4) was used the average probability of the miscoded group was 0.40 (0.66) and 0.007 (0.006) for the correctly coded observations.

In real data set application, the miscoded observations will be unknown and a reliable cutoff probability is desired. Table 3 presents the percent of misclassified individuals correctly identified based on two classification probabilities. We first applied a hard cut off probability set at 0.5. At this limit, our proposed method (M3) was able to account for 27 and 24% of the misclassified individuals based on D1 and D3, respectively (Table 4). This is mostly due to the fact that setting such a strict cutoff does not allow for much variation around the threshold. In this case individuals with probabilities very close to 0.5 were not accounted for. As the odds ratios increased, even with the strict cutoff applied, 95 and 90% of the misclassified groups were identified for D2 and D4, respectively (Table 4). In order to relax the restrictions of a hard cut off probability, a soft classification approach was used where observations are declared to be misclassified if they exceeded a heuristically determined threshold. In this study, the threshold was set based on the overall mean of the probabilities of being misclassified over the entire dataset plus two standard deviations. Both moderate scenarios, D1 and D3, showed better results compared to the strict cutoff as M3 correctly identified 94 and 79% of the misclassified observations. As the odds ratios increase, the genetic differences between cases and controls become more distinguishable allowing for better detection. This can be seen when the extreme case scenarios are used as 99% of the misclassified individuals were identified for D2 and 97% for D4 (Table 4). Furthermore, across all four scenarios and both cutoff probabilities, no correctly classified observation has a misclassification probability exceeding the cut off threshold and therefore was not incorrectly switched (Table 4). This further shows a tendency for misclassified individuals having higher probabilities compared to the correctly coded groups. It is worth mentioning that this study was limited to the situation where a misclassification probability was assumed to be the same in cases and controls which is not always the case based on real human disease data. In fact, our follow up study (results not shown) has investigated the performance of the proposed method with varying misclassification probabilities for cases and controls. The results were similar in trend and magnitude to those observed in this study. Additionally, the model used at the liability scale in this study is rather simple as it account only for additive effects of relatively small set of SNPs. In real GWAS applications, the number of SNPs is often much larger than the number of observations and, thus, some of the priors used in this study will not be appropriate. Hierarchical generalized linear mixed models [47,48] provide a flexible and robust alternative. In fact, an elegant procedure has been adopted [48] for accommodating individual variant (SNPs) effects as well as group (i.e. gene) effects. In the presence of epistatic effects, a study [49] presented an empirical Bayesian regression approach for accommodating these effects using logistic regression. In all cases, either due to the increase in the number of variant effects or the assumption of a more complex genetic model (presence of epistatic effects), our approach will easily accommodate these modifications through the adjustment of the linear model assumed at the liability scale in our study and the appropriate specification of prior distributions and their hyper-parameters following the above mentioned studies. Finally, our study was limited to only one binary trait and it will be interesting to evaluate its performance in presence of multiple binary traits or multinomial responses.

**Table 4:** Percent of misclassified individuals correctly identified based on two cutoff probabilities across the four simulation scenarios

	**D1**		**D2**		**D3**		**D4**	
	Misclass^2^	Correct	Misclass	Correct	Misclass	Correct	Misclass	Correct
Hard^1^	0.27	0	0.95	0	0.24	0	0.90	0
Soft	0.94	0	0.99	0	0.79	0	0.97	0

^1^Hard: cut off probability was set at 0.5. Soft: cut off probability was equal to the overall mean of the probabilities of being misclassified over the entire dataset plus two standard deviations; ^2^Misclass: individuals which were misclassified. Correct: Correctly coded individuals.

## Conclusions

Misclassification of discrete responses has been shown to occur often in datasets and has proven to be difficult and often expensive to resolve before analyses are run. Ignoring misclassified observations increases the uncertainty of significant associations that may be found leading to inaccurate estimates of the effects of relevant genetic variants. The method proposed in this study was capable of identifying miscoded observations, and in fact these individuals were distinguished from the correctly coded set and were detected at higher probabilities over all four simulation scenarios. This is essential as it shows the capability of our algorithm to maintain its superior performance across different levels of misclassification as well as different odds ratios of the influential SNPs.

More notably, our method was able to estimate SNP effects with higher accuracy compared to estimation using the “noisy” data. Running analyses on data that do not account for potential misclassification of binary responses, such as M2 in this study, will lead to non-replicative results as well as causing an inaccurate estimation of the effect of polymorphisms which can be correlated to the disease of interest. This severely reduces the power of the study. For instance, it was determined that conducting a study on 5000 cases and 5000 controls with 20% of the samples being misdiagnosed has the power equivalent to only 64% of the actual sample size [7]. Implementing our proposed method provides the ability to produce more reliable estimates of SNP effects increasing predictive power and reducing any bias that may have been caused by misclassification. Our results suggested that the proposed method is effective for implementation of association studies for binary responses subject to misclassification.

## Abbreviations

SNP: Single nucleotide polymorphism; OR: Odds ratios; GWAS: Genome-wide association studies; PM: Posterior mean; HPD95%: High posterior density 95% interval.

## Competing interests

The authors declare that they have no competing interests.

## Authors’ contributions

The first author (SS) has contributed to all phases of the study including data simulation, analysis, discussion of results and drafting. EHH helped with data analysis and drafting. NF participated in the development of the general idea of the study and drafting. RR has participated and supervised all phases of the project. All authors read and approved the final manuscript.
